# Supplementary material for: Avidity observed between a bivalent inhibitor and an enzyme monomer with a single active site
Source: PLoS One. 2021 Nov 30;16(11):e0249616. doi: 10.1371/journal.pone.0249616 (PMC8631645; doi:10.1371/journal.pone.0249616)
Supplement: S1 Raw images — This figure shows the original gels presented in Figs 1E and 2C, respectively. (DOCX) [file pone.0249616.s001.docx]

**S1_raw_images:** 15% SDS-PAGE analysis of the purified monomeric APPI fraction (**A**) and comparison of purified monomeric APPI with dimeric APPI before and after purification (**B**). This figure shows the original gels presented in Fig. 1E and 2C, respectively.


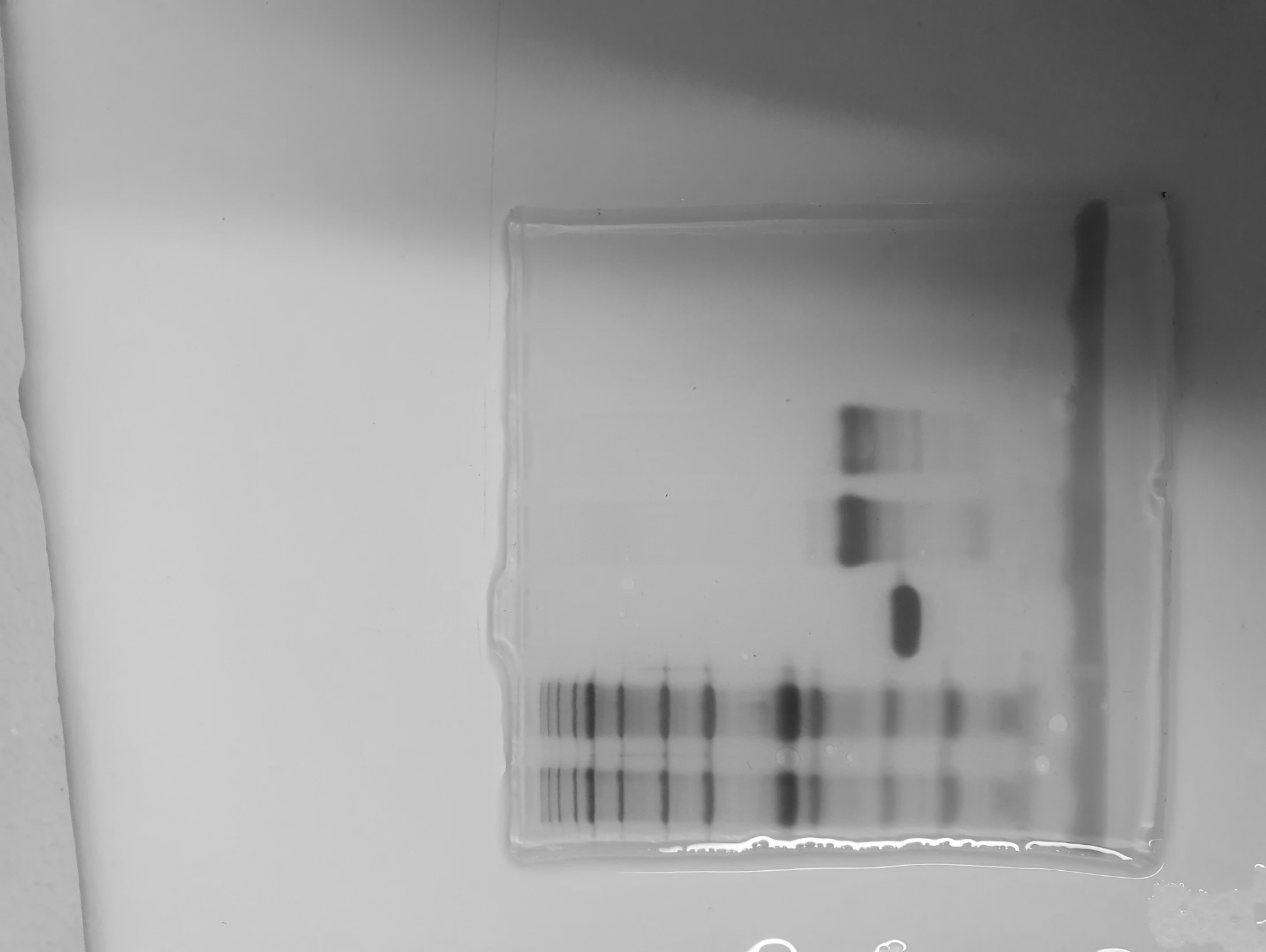


**10 kDa**

**15 kDa**

**20 kDa**

**25 kDa**

**Monomer**

**Dimer**

**before**

**Dimer**

**after**


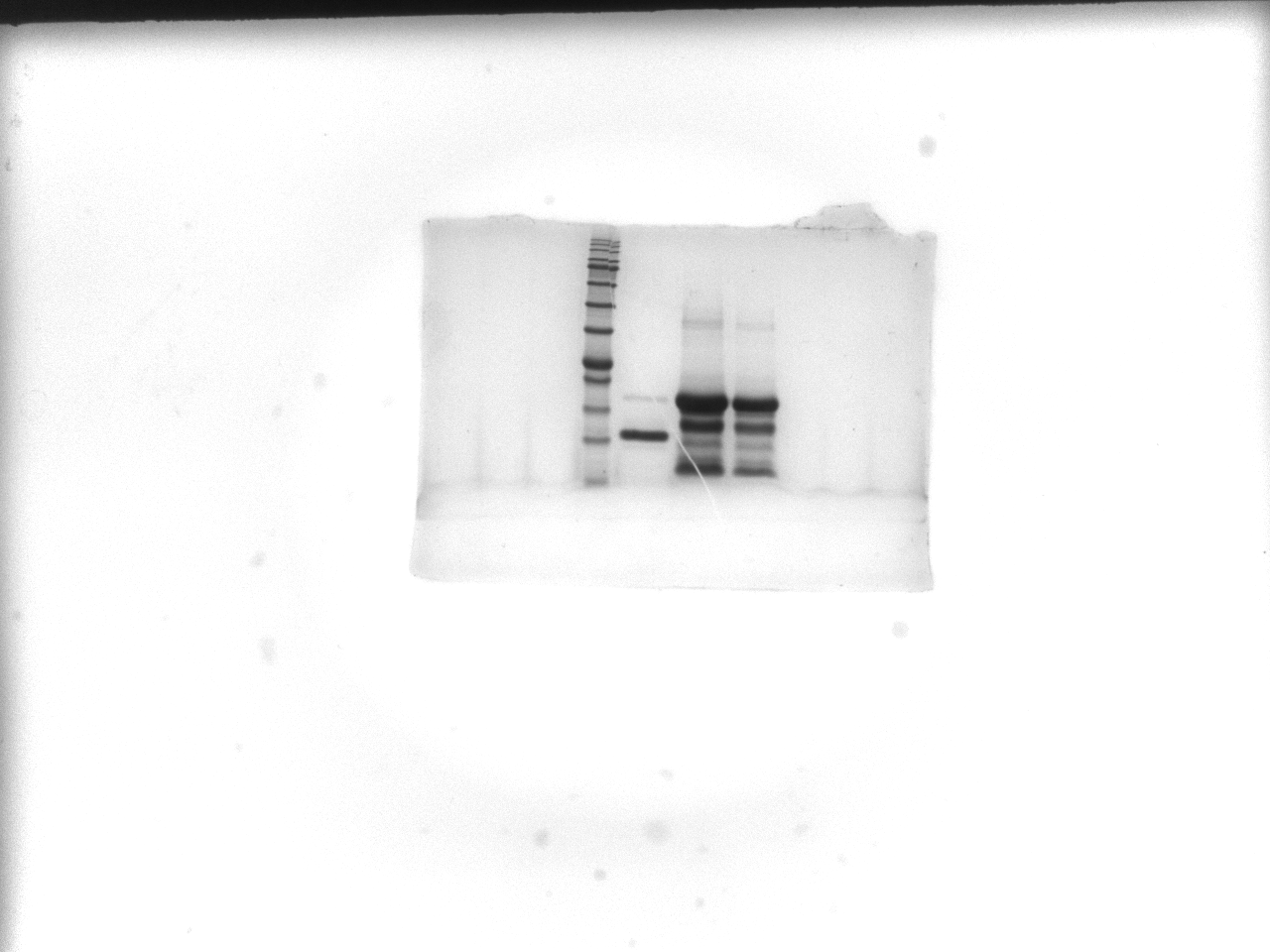


**10 kDa**

**15 kDa**

**20 kDa**

**APPI monomer**

**A**

**B**
