# Supplementary material for: Avidity observed between a bivalent inhibitor and an enzyme monomer with a single active site
Source: PLoS One. 2021 Nov 30;16(11):e0249616. doi: 10.1371/journal.pone.0249616 (PMC8631645; doi:10.1371/journal.pone.0249616)
Supplement: S1 File — (DOCX) [file pone.0249616.s003.docx]

**Supplementary Information**

**Supplementary Methods**

**Derivation of Eq. (15)**

Here we provide a step-by-step derivation of Eq. (15) reported in the main text. In order to derive Eq. (15), we use Eq. (3), Eq. (6), Eq. (10), combined with Eq. (14). We now rewrite these equations for convenience of the derivation:

$\frac{[E][S]}{[ES]}=K_{M}$ Eq. S1

$\frac{[E][I]}{[EI]}=\mathbf{K}_{I}$ Eq. S2

$\frac{{[E]}^{2}[I]}{[(2E)I]}=\mathbf{K}_{2I}^{2}$ Eq. S3

$\left[ E \right]+\left[ ES \right]+[EI]+2\left[ (2E)I \right]=E_{tot}$ Eq. S4

where all the parameters are defined in the main text. First, we express [*E*] from Eq. S1:

$[E]=K_{M}\frac{[ES]}{[S]}$ Eq. S5

Second, we substitute the expression for [*E*], Eq. S5, into Eq. S2, Eq. S3, and Eq. S4, respectively. As a result of this substitution we obtain for Eq. S2:

$[EI]=\frac{K_{M}[ES][I]}{[S]\mathbf{K}_{I}}$ Eq. S6

for Eq. S3:

$\frac{K_{M}^{2}{[ES]}^{2}[I]}{{[S]}^{2}[(2E)I]}=\mathbf{K}_{2I}^{2}$ Eq. S7

and for Eq. S4:

$\left[ ES \right](1+\frac{K_{M}}{[S]})+[EI]+2\left[ (2E)I \right]=E_{tot}$ Eq. S8

Next, we substitute, [*EI*] from Eq. S6 into Eq. S8, and then express [(2*E*)*I*] from the resulting expression:

$\left[ (2E)I \right]=\frac{1}{2}\left( E_{tot}-[ES]\left( 1+\frac{{\alpha K}_{M}}{[S]} \right) \right)$ Eq. S9

where

$\alpha=1+\frac{[I]}{\mathbf{K}_{I}}$ Eq. S10

Finally, substituting [(2*E*)*I*] from Eq. S9 into Eq. S7, we obtain:

${[ES]}^{2} \frac{\boldsymbol{2}[I]}{\mathbf{K}_{2I}^{2}}\left( \frac{K_{M}}{[S]} \right)^{2}+\left[ ES \right]\left( 1+\frac{K_{M}}{\left[ S \right]}\alpha\right)-E_{tot}=0$ Eq. S11

The last equation, Eq. S11, represents Eq. (15) of the main text.

**Supplementary Figures**

**Figure S1: Strategy for inserting dimeric APPI into *P. pastoris***. **(A)** Generating the dimeric APPI gene construct in pPic9k plasmids by the gene assembly method. **(B)** Gel electrophoresis of PCR products on 1% agarose gel. The red arrows indicate the monomeric and dimeric APPI, whereas the upper band indicates the presence of an alcohol oxidase gene which is also regulated by the AOX promoter. **(C)** Representative chromatogram of the gene sequence encoding APPI dimer. The horizontal black arrow indicates the insertion position of a GGGGS×3 linker gene.

**Figure S2: Mass-spectrometry spectra**. Spectra of **(A)** monomeric and **(B**) dimeric APPI, obtained using Matrix-Assisted Laser Desorption/Ionization-Time of Flight (MALDI-TOF) analysis.

|  | **APPI monomer** | **APPI dimer** |
| --- | --- | --- |
| **Absorbance (M)** | (120.0±2.0)×10^-6^ | (16.4±1.1)×10^-6^ |
| **Activity (M)** | (107.4±3.0)×10^-6^ | (27.2±1.0)×10^-6^ (M inhibitory units) |
|  |  | (13.6±0.5)×10^-6^ (M concentration assuming 1:2 stoichiometry) |

**Table S1: Protein concentration determination.** APPI variant concentrations were determined using absorbance at 280 nm and trypsin titration analysis *vs.* bovine trypsin, when assuming 1:1 binding stoichiometry for APPI monomer:trypsin, and 1:1 or 1:2 binding stoichiometry for APPI dimer:trypsin.
