# Supplementary material for: Avidity observed between a bivalent inhibitor and an enzyme monomer with a single active site
Source: PLoS One. 2021 Nov 30;16(11):e0249616. doi: 10.1371/journal.pone.0249616 (PMC8631645; doi:10.1371/journal.pone.0249616)
Supplement: Attachment — Submitted filename: plosonecomments.pdf [file pone.0249616.s004.pdf]

The Papo group examines here if a bivalent inhibitor displays avidity towards its monomeric enzyme target. Understanding avidity, or as it is known in chemistry, multivalency, is one of the most important and interesting challenges yet to be fully solved when it comes to understanding intermolecular interactions. It is, as the authors point out, of particular importance when it comes to various biological process as well as in other fields including medical chemistry and nanoscience.

The key claim of this paper seems to be that the authors have uncovered a new insight into avidity through a very simple system; a bivalent inhibitor interacting with its monomer enzyme target.

I am really not certain if this is correct and whether the paper should be published will in my opinion come down to if the authors can make a more convincing case for this claim and make the connections of this work clearer with the current understanding of the field of avidity/multivalency, particularly from within the field of supramolecular chemistry. I will expand on these concerns in more details below:

1. Part of the problem with this work is that researchers in biology/biophysics and those in (supramolecular) chemistry use a completely different language about what are essentially the same things – the (almost) synonyms of avidity and multivalency being a prime example. Usually what happens then is that researchers will go to extreme lengths to make it appear that there are some real differences between these terms and again, in the literature there are some very interesting examples of authors trying to make it so that avidity and multivalency are different things when there is little substance behind these terms. With this in mind, I would like to start to suggest to the authors that they have good look at the chemistry literature on multivalency, e.g. from authors such as Gianfranco Ercolani, George Whitesides, Pall Thordarson and Harry Anderson to mentioned a just few (see c.f. Angew Chem 1998, 37, 2754, J. Phys. Chem. B, 2007, 111, 12195, Angew. Chem. 2009, 48, 7488, Angew. Chem., 2011, 50, 1762, Chem. Soc. Rev. 2017, 46, 2622, Bioconjugate Chem. 2019, 30, 503). This might obviously have some impact on their introduction to the topic.

2. An often-mentioned topic by the authors/papers mentioned in 1 is that when it comes to "multivalent" binding, statistical factors need to be taken into account. As discussed also in details in Chem Soc Rev 2011, 40, 1305, the 2 to 1 interaction is often used as an archetypal example. Accordingly, care needs to be taken when discussing a 2:1 (or 1:2 – the difference really is only about which partner in the interaction is defined as the first one) interactions, do the authors mean, e.g., the microscopic ($\boldsymbol{K_\mathrm{m}}$) or macroscopic ($\boldsymbol{K}$) binding constants, or do they mean the overall binding constants (which in chemistry is sometimes shown as β to avoid confusion) in their description of say, $\mathbf{K}_I$ in Figure 5/ eq 4 which is shown there in the power of 2: $\mathbf{K}_I^2$? By the looks of it , $\mathbf{K}_I$ is really the inverse microscopic binding constant (here it does not help that chemist usually use association constants while biologists prefer dissociation constants which are simply the inverse of the former) for each stepwise interactions and, additionally, the authors have made the assumptions to use the language from Chem Soc Rev 2011, 40, 1305 that $\mathbf{K}_I = \boldsymbol{K_\mathrm{1m}} = \boldsymbol{K_\mathrm{2m}}$, i.e. the stepwise interactions have no cooperativity. It does not help here that in Eq 2, $\mathbf{K}_I$ appears to be what in chemistry would be called the overall binding constant with the units M^{-2}.

3. Continuing with the equations: Fig 5 / eq 4 has $\mathbf{K}_I^{\mathbf{2}} = k_{i'}/k_i$ in which the two rate constants come from eq 3. But this does not make a lot of sense? This must be a stepwise process (here <-> equilibrium arrows:

E + I <-> EI   step 1, rate constants are k_1 and k_{-1}

EI + E <-> EI   step 2, rate constants are k_2 and k_{-2}

Hence Eq 4 seems incorrect. This is also evident if one considers that for Eq 4 to be correct, $k_{i'}/k_i$ must have the units M^2.

4. Continuing to Eq 5, I cannot see how it was obtained from Eq 3. Whether my concerns about Eq 3 above are correct or not, the authors need to give more details on this derivation, at least in SI (write it out in full if necessary).

5. Figure 6 – which non-linear models / equations? This is really not clear at all. Again, more details, and ideally, raw data file etc should be included as SI.

6. Eq 8 on page 22 and related discussion earlier in the paper: Why does the bivalent inhibitor not bind to two trypsin (but only with mesotrypsin)? Or is this not correctly understood.

7. Eq 9 seems to be the main method to measure inhibition. But isn't this very risky? As the Graphpad manual points out this should not be used for tight binding, rather, using the Morrison equation:

GraphPad Prism 9 Curve Fitting Guide - Equation: Tight inhibition (Morrison equation)

Now, a companying paper Microsoft PowerPoint - 1305-arqule-002.ppt (biokin.com) explains this issue in more details.

But on reading about the Morrison Equation, it seems it is a quadratic question that is strongly related to those used to find free ligand concentration in a typical 1:1 equilibria. If that is so, there should be a cubic equation equivalent of the Morrison equation that would be related to the cubic one for 1:2 or 2:1 equilibria in Chem Soc Rev 2011, 40, 1305?

8. Somewhat related to 7 is that the authors say on page 23 that there is excess of the inhibitor that argue therefore I think that the "tight binding" models might not be necessary. But based on experience from supramolecular chemistry, even with such excess this is a dangerous assumption to make at any point for 1:2 and 2:1 equilibria.

9. And on the topic of concentration – is the concentration of the dimer inhibitor reported in various plots etc, the concentration of the molecule or the concentration of binding loops? The latter would be 2x the former. I mentioned this because IF the assumption is made that there is no cooperativity between the first and second enzyme binding to the inhibitor, then it should be possible to model the system as a simple 1:1 system provides the 2x inhibitor concentration is used in all the data analysis.

11. It would also help if the authors simply included as much as possible of their raw data and fitting program(s) as an attached (could be deposited on github or figshare).

10. In conclusions, this paper appears to be an important contribution to understand avidity / multivalency / cooperativity better. But a quite a number of issues need to be cleared up first and the data analysis explained in enough details to make it both reproducible and unambiguous in terms of links to other related work, particularly in chemistry.
