# Supplementary material for: Avidity observed between a bivalent inhibitor and an enzyme monomer with a single active site
Source: PLoS One. 2021 Nov 30;16(11):e0249616. doi: 10.1371/journal.pone.0249616 (PMC8631645; doi:10.1371/journal.pone.0249616)
Supplement: Attachment — Submitted filename: Response to Reviewers.docx [file pone.0249616.s006.docx]

**Reviewer #2:** I thank the authors for the revision of their manuscript and for having by and large addressed all my concerns and questions. I have only one remaining suggestion, and the editor does not need to send the paper back to me should the authors do this:

The derivation of what is equation 15 is still not clear. Yes, it is obtained by combining Eq 3, 6 and 10. But exactly how that leads to 15 is a bit unclear. Perhaps include a more detailed step-by-step derivation in the SI?

**Answer:** We thank the reviewer for the comment and added a more detailed step-by-step derivation in the SI section.
